# Microfabricated Physiological Models for In Vitro Drug Screening Applications

**DOI:** 10.3390/mi7120233

**Published:** 2016-12-15

**Authors:** Giovanni Stefano Ugolini, Daniela Cruz-Moreira, Roberta Visone, Alberto Redaelli, Marco Rasponi

**Affiliations:** Department of Electronics, Information and Bioengineering, Politecnico di Milano, Milan 20133, Italy; giovannistefano.ugolini@polimi.it (G.S.U.); daniela.cruzmoreira@polimi.it (D.C.-M.); roberta.visone@polimi.it (R.V.); alberto.redaelli@polimi.it (A.R.)

**Keywords:** microfluidics, drug screening, physiological models, organ-on-chips

## Abstract

Microfluidics and microfabrication have recently been established as promising tools for developing a new generation of in vitro cell culture microdevices. The reduced amounts of reagents employed within cell culture microdevices make them particularly appealing to drug screening processes. In addition, latest advancements in recreating physiologically relevant cell culture conditions within microfabricated devices encourage the idea of using such advanced biological models in improving the screening of drug candidates prior to in vivo testing. In this review, we discuss microfluidics-based models employed for chemical/drug screening and the strategies to mimic various physiological conditions: fine control of 3D extra-cellular matrix environment, physical and chemical cues provided to cells and organization of co-cultures. We also envision future directions for achieving multi-organ microfluidic devices.

## 1. Introduction

Discovery of new therapeutics is an essential endeavor, encompassing several branches of science from chemistry to biology and representing a remarkable economic burden worldwide. The process of taking a candidate drug compound from discovery to the market is estimated to cost from hundreds of millions to billions of US dollars to the industry [1,2]. A key step in this process is obtaining evidence of drug safety, efficacy and mechanism of action on representative biological models (pre-clinical studies) before testing candidate compounds on humans [3].

The information obtained in pre-clinical studies provides data on the effects that candidate drug compounds have on cellular-scale systems or organism-scale systems. In vitro studies are conducted by supplying candidate compounds to relevant cell culture models (e.g., hepatocytes for hepatotoxicity evaluation) [4], however, this testing alone is not reliable enough to predict biological plausibility, active dosing ranges, safety and targeting efficiency [5]. In vivo animal testing is currently the most employed pre-clinical tool for determining scientific grounds for further development of a candidate drug compound. Indeed, animal models are a unique source of fundamental in vivo information on systemic organism-level drug response, not otherwise achievable with artificial in vitro models [6]. After pre-clinical in vitro and in vivo studies, candidate drug compounds that meet efficiency and safety criteria are further evaluated through human clinical trials.

Ideally, an optimal screening system should allow identification of most failures during early-stage experiments. Nevertheless, the drug development process is currently troubled by high attrition rates as most candidate compounds fail in late-stage screening steps, denoting a greater economic burden for the process [7]. Indeed, the greatest fraction of drug development expenses is invested in clinical trials, yet it is estimated that chances of clinical trial success are currently about 10% and the total time-to-market of a candidate drug compound is estimated to be around 12 years [8,9]. In light of this, it is not surprising that doubts have been cast on the reliability of pre-clinical assessments [10]. It is known that, as for in vivo studies, immunological, genetic and cellular differences exist between animals and humans, thus making animal models poor predictors of the whole spectrum of physiological responses that may occur when humans are administered a drug compound [11,12]. In addition, current in vitro screenings are conducted on bidimensional polystyrene-based vessels, regarded to as standard cell culture systems. These are considered to have poor similarity to physiological environments in terms of dimensionality, physical and chemical environment, thus retaining limited potential for efficient and reliable screening of drug-induced effects. An increasing need for efficient and predictive pre-clinical studies is therefore clear and improving current approaches to drug screening is paramount for rationalizing drug development process and avoid costly late-stage failures of novel drug compounds.

One of the strategies that attracted interest and expectations is increasing the efficiency of pre-clinical in vitro studies by employing culture systems that better recapitulate physiological microenvironments. Mimicking specific characteristics (spatial architecture of cellular actors, physical environment, and chemical stimulation) of drug-targeted in vivo districts (organs, tissues or biological functional units) may indeed hold potential for performing in vitro drug testing that is more specifically tailored to the target physiological structures, with superior efficiency in predicting drug behavior. Among technical solutions for engineering advanced, physiologically relevant in vitro culture models, microfabrication and microfluidics have emerged in the last decade as promising techniques for basic and translational research [13,14]. Indeed, microfabricated systems feature small loading volumes (of the order of microliters), advantageous for reducing amounts of drug compounds and cells used in assays, increasing the chances of employing cells directly isolated from humans. In addition, a greater control of physico-chemical stimulation and of spatial architecture is possible within microfabricated channels, broadening the potential for better mimicking in vivo structures, improving drug-screening processes and reducing the number of animals used in in vivo drug testing steps.

The last decade saw the rise of bio-inspired microfabrication technologies, with a large number of studies reporting advanced biological models cultured by means of microdevices. The current literature on microdevices and microfabrication techniques dedicated to the culturing of advance models of in vivo biological structures has been widely reviewed elsewhere [15,16,17,18,19,20]. In an attempt to contextualize the potential of micron-scale approaches as tools for advanced pre-clinical drug screenings, we here provide an outlook on microfabrication solutions for mimicking physiological environments, emphasizing studies that employed such techniques for analyzing the response of biological entities to chemicals and drug stimulation. We summarized the literature by separately discussing different relevant in vivo biological structures (organs or tissues) and providing a final perspective opinion on future modular approaches for incorporating multiple tissue or organ units within a single screening microdevice (body-on-chip).

## 2. Liver

The liver is the first organ perfused by compounds absorbed in the digestive tract and the main metabolizing organ in humans. Liver cells, specifically hepatocytes, are organized into functional units named lobules and are responsible for breakdown of drug compounds. Hepatocytes therefore represent a fundamental in vitro model largely employed in pre-clinical screenings for evaluating drug toxicity, metabolic behavior and drug transporters function. Typical assays for in vitro pre-clinical screenings of hepatic drug response involve massive parallelization of standard hepatocytes cell cultures (high-throughput screening) and analysis of metabolic responses and hepatotoxic effects [21]. For instance, levels of Cytochrome P450 enzymes (CYP) represent a gold-standard parameter for evaluating hepatocytes metabolism [22]. However, hepatocytes cultured within standard conditions present phenotypic and metabolic differences with in vivo hepatocytes. It was shown that levels of CYP enzymes are dissimilar to the expected levels found in vivo [23]. What is more, they often result unaffected by chemical stimulation in vitro [23].

The dramatically different environmental conditions occurring in in vitro systems are likely responsible for these discrepancies. For instance, microenvironmental cell–cell and cell–extracellular matrix (ECM) interactions are more precisely reconstructed within 3D hydrogel-based or spheroid-based cellular constructs, rather than in bidimensional culture platforms [24,25]. Although these approaches are achievable within hanging drops or multi-well plates, microfabricated platforms exhibit a higher control in handling small scale fluid flow and are particularly suitable for forming, culturing and chemically addressing 3D cellular constructs [26,27]. Among the examples of 3D hepatic cultures employed for drug screening that we found in current literature [28,29,30,31,32,33], Au et al. developed a digital microfluidic device for generating and individually addressing 3D hydrogel-based hepatic microtissues within micro-droplets. The authors employed this microplatform to perform enzymatic assays by treating constructs with inducers or inhibitors of CYP activity and hepatotoxicity screenings by testing the response of microtissues to acetaminophen. Interestingly, differences in enzymatic activity were not detectable after treatment of standard monolayers of hepatocytes while significant alterations of enzymatic activity were reported in 3D constructs cultured within the microfluidic device [29].

**Figure 1:**
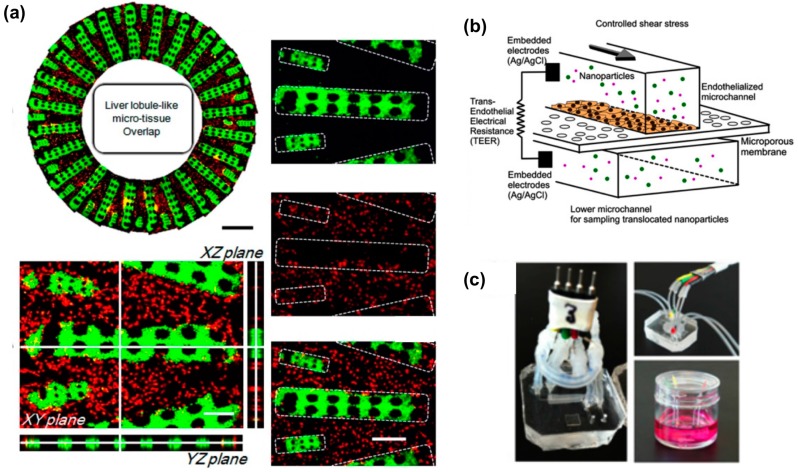
(**a**) Geometrical 3D arrangement of a liver lobule-on-a-chip. An hydrogel patterning technique is employed by Ma et al. to obtain radially distributed hepatocytes (green) in a network of endothelial cells (red). Adapted from [42] with permission; (**b**) 3D sketch of a blood–brain barrier microdevice model with upper compartment hosting endothelial monolayer culture on a microporous membrane and lower compartment for collection of transported nanoparticles. Electrical measurements across the endothelial monolayer provide information on barrier integrity; (**c**) Pictures of blood–brain barrier microdevices with fluidic and electical connections (adapted from [52] with permission).

Mimicking the architecture of the liver lobules in terms of cell types contributions is another valuable strategy towards engineering an in vivo-like liver model for drug screening. Indeed, co-culturing hepatocytes with other relevant liver cell types increases the performance of in vitro hepatic models [34,35]. The most relevant cell types employed to interact with hepatocytes are endothelial cells, non-parenchymal cells such as fibroblasts [36,37], Kupffer cells [38] and hepatic stellate cells [39]. We report a number of multi-culture studies conducted in microdevices for hepatic drug screening [39,40,41,42]. In a recent work, Ma et al. described a microdevice organized to mimick the distribution of hepatocytes and endothelial cells in the liver lobule. The authors described a technique for confining radially distributed hepatocytes embedded in 3D hydrogels, intertwined with endothelial cells in a hepatic sinusoid-like structure. Hepatocytes cultured within the microplatform exhibited higher basal CYP activity compared to control hepatocytes cultured in standard conditions (2D hepatocytes alone or hepatocytes alone embedded in 3D hydrogels). This in vitro platform was then employed to perform drug–drug interaction studies between omeprazole, rifampicin, ciprofloxacin and probenecid. However, only cell viability assays were conducted after drug incubation and the potential of the model in assessing metabolic and enzymatic activity was not demonstrated [42]. In an earlier work by Khetain and Bhatia, hepatocytes and fibroblasts were co-cultured by means of microfabricated polydimethylsiloxane (PDMS) stencils (Figure 1) [41]. The utility of co-culturing was elegantly demonstrated by showing maintained metabolic activities (CYP enzymes activity and phase II metabolism) up to Day 20, while mono-cultures of hepatocytes exhibited marked loss of activity at Day 11. In addition, the authors provided a relevant set of hepatotoxicity data by characterizing a large panel of compounds in terms of TC_50_ (concentration of compound that leads to a 50% decrease in mitochondrial activity after acute exposure) and modulation of CYP activity.

Another key physiological aspect that is often overlooked in standard cell cultures is the constant perfusion of cultured cells. Blood flow constantly perfuses hepatocytes in vivo, supplying nutrients and removing waste. Nevertheless, standard cell cultures fail to reproduce this aspect in vitro: the static volume of culture medium typically supplied to cells in standard culture vessels leads to nonsteady-state conditions with nutrients diminishing and waste increasing over time in an uncontrolled manner. Constant perfusion cell culture systems, on the other hand, are able to precisely control steady concentration profiles of nutrients and drug molecules to cultured cells. High resolutions obtained with standard microfabrication techniques allow a remarkable control over fluidic quantities within microfabricated devices. In the field of liver models for drug screening, several microdevice-based liver models employed flow-based systems [43,44,45,46,47,48,49,50]. Leclerc group has successfully developed a constant perfusion microdevice where hepatocytes exhibited higher functional parameters compared to standard Petri dishes [43]. Subsequently, this device was extensively employed to characterize acetaminophen hepatoxicity [45] and to predict the in vivo clearances of a panel of seven drug compounds in rats [44]. Notably, while most of the previously described approaches employed HepG2, a murine cell line known to have inherently low CYP activity, in some of these studies the authors successfully employed human primary hepatocytes therefore moving towards a more accurate mimic of the human hepatic environment. Further, Lee et al. coupled the benefits of applying constant flowrate to the improved physiological mimicry of an endothelial-hepatocytes co-culture. In the developed microdevice, hepatocytes are seeded in a central culture channel, lined by narrow confining structures that separate high-density hepatocytes from a flow channel where endothelial cells are seeded. This hepatic sinusoid-like device was validated with the hepatotoxic drug diclofenac [48]. Finally, it is worth noting that microfluidic approaches to liver drug-response studies have generated relevant commercial devices from several companies (CellASIC, HμREL, and Organovo) tailored to this application [51].

## 3. Central Nervous System

Central Nervous System (CNS) disorders are known to represent a severe threat to the organism mainly due to the limited regenerative potential of brain tissue, limited immune response and difficulty in CNS targeting by drug molecules. Due to the complexity of the disorders, the largely unknown anatomy and biology of the brain and the physiological barriers protecting the CNS (blood–brain barrier), fewer approved drugs are targeted at the CNS compared to other therapeutic areas. To date, major CNS disorders such as Parkinson’s disease, Huntington’s disease or multiple sclerosis still lack efficient pharmacological treatment [53].

In order to evaluate drug efficacy, safety and mechanism of action, it is important to recreate aspects of neuronal physiology in vitro such as the formation of neurites or axonal protrusions connecting neurons with each other in a functional network. This phenomenon is key for reproducing in vivo-like brain cultures and results regulated by complex interplays of chemical and physical cues. Microfluidic devices have been employed in literature to assess neurite and axonal formation under chemical stimulation in the form of controlled gradients of neurotrophic factors [54,55,56,57,58,59,60,61,62]. In addition, microstructured topologies have been employed to guide axonal growth for subsequent drug screening studies [63,64,65,66,67]. In a work by Taylor et al., a microfabricated platform was employed to seed neurons in a somal compartment connected to an axonal compartment by multiple branched microgrooves. Axonal growth was observed after seven days, guided by the presence of the microgrooves. The authors then induced axonal injury by vacuum aspiration and tested the regenerative efficiency of brain-derived neurotrophic factor and neurotrophin-3 [63].

Another crucial aspect of neuronal physiology is the electrical activity of neuronal networks. Direct reading of electrophysiological signals generated by cultured neurons is relevant for studying responses and mechanism of action of potential CNS drugs [68]. While micro-electrode arrays (MEA) represent standard tools for recording electrical activity of cultured cells, their potential can be further broadened by microfluidic techniques. For instance, MEAs have been integrated with microfluidic devices for improving spatial control of chemical stimulations [69,70,71,72] or optical visibility [73] of cultures subject to drug screening.

Pre-clinical drug screenings of CNS-targeting drug formulation are not only addressed at evaluating safety and efficacy in restoring physiological neuronal functions and synaptic activity. An even greater challenge exists in the effective delivery of candidate drugs to the pathological CNS site of interest. While peripheral blood vessels or capillaries normally exchange nutrients and small molecules with surrounding tissues, CNS endothelial cells form tighter junctions in vessel walls and exhibit an overall reduced permeability to small molecules. This peculiar structure of CNS vessels is named blood–brain barrier (BBB) and has tremendous implications for CNS drugs in terms of targeting efficiency as 98% of small molecules do not cross the BBB [74,75]. Since the bloodstream is the most relevant delivery route for drugs targeted at the CNS, a major step in CNS drug development is to engineer and to evaluate drug capability of crossing the BBB [76]. A number of microfluidic devices showed how to improve standard Transwell-based in vitro models of BBB transport. A class of 2D microfluidic devices where microfluidically-controlled tight endothelial cells monolayers are cultured on porous substrates [52,77,78,79,80] has indeed shown advancements such as controlled biomimetic physical stimulation of cells (shear stress), dramatically reduced amounts of drug molecules used in experiments and integration of monolayer tightness monitoring systems based on electrical resistance measurements. In a work by Kim et al., a microfluidic device was employed to assess translocation of nanoparticle-based drug formulations through an endothelial monolayer monitored by means of integrated electrodes for trans-endothelial electrical resistance (TEER) measurements and chemically stimulated with inflammatory factors (Figure 1). Results of nanoparticle permeability were then compared to in vivo animal models of atherosclerosis [52].

Alongside microdevices based on bidimensional cultures of endothelial BBB monolayers, strategies have recently emerged for culturing 3D structures resembling the organization of the physiological BBB within microfabricated platforms and performing drug screening experiments. Brown et al., described a multi-layer PDMS microplatform with a microporous membrane sandwiched between two microfabricated channels: the bottom channel was dedicated to endothelial cell culture and the top channel to the injection of a collagen-based 3D hydrogel embedding neurons and astrocytes [81]. The formation of a BBB-like endothelial barrier was investigated by measuring TEER and determining the diffusion of fluorescein isothiocyanate (FITC)—dextran with and without chemical stimulation disrupting the BBB. In addition, studies report the fabrication of vessel-like structures where endothelial cells line a tubular, hollow 3D structure [82,83,84]. Herland et al., developed a 3D model of BBB featuring a tubular hollow collagen gel formed by viscous fingering and embedding human astrocytes. This lumen structure was filled with a cell solution of human endothelial cells and pericytes. The authors estimated apparent permeability of the endothelial monolayers to FITC-dextran and stimulated the constructs with an inflammatory cytokine (tumor necrosis factor alpha). Results of these experiments provide insights into the contribution of astrocytes and pericytes to neuroinflammation and demonstrate that the inflammatory responses of the engineered BBB more closely mimics those observed in vivo compared to standard culture systems.

## 4. Heart

Cardiotoxicity is a main issue in drug development, frequently undetected during initial phases of drug screening and showing great differences depending on cardiac pathology and genetic variants [85]. Microfluidic devices have become a valuable tool for studying cardiac cells and the effects of heart-targeted drugs or of drugs directed to other diseases and organ systems. The main advancements brought by microfabricated platforms and appealing to cardiac biology include: reduced number of cells employed in assays, capability to subject cultured cells to relevant cardiac-like stimulations (mechanical [86,87] or electrical [88] cues) and capability to couple contractility and electrical activity measurements [89,90,91,92].

**Figure 2:**
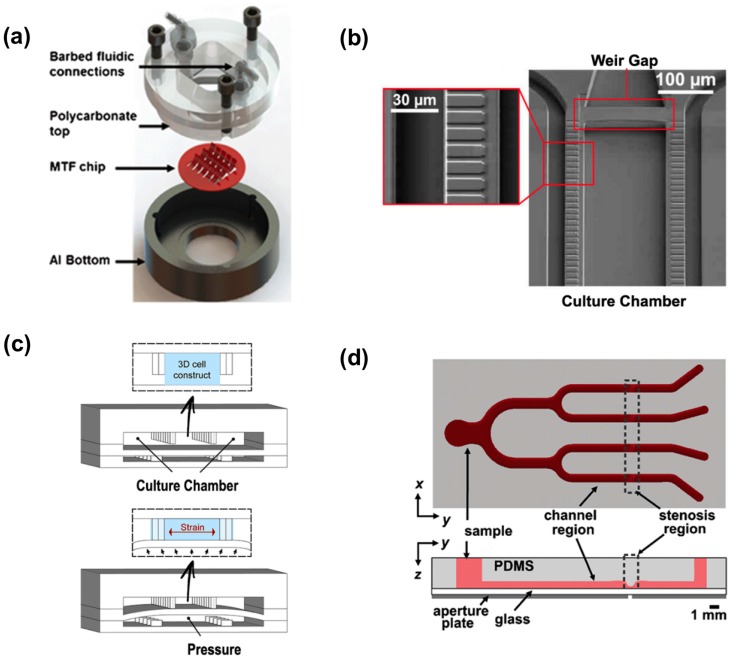
Cardiovascular organs-on-a-chip. (**a**) Exploded view of heart-on-a-chip device. Adapted from [96] with permission from The Royal Society of Chemistry; (**b**) Scanning electron micrograph of the microphysiological system. Red rectangular boxes show the 2 mm endothelial-like barrier and the weir gap. Adapted with permission from [103]; (**c**) Schematic of the 3D beating heart-on-a-chip microdevice. The polydimethylsiloxane (PDMS) membrane between compartments deforms, compressing the 3D cell construct. Adapted from [87] with permission from The Royal Society of Chemistry; (**d**) Schematic showing top and side view of the microfluidic device for inducing platelet aggregation at four distinct high shear stenotic regions. Adapted with permission from [104].

Microfluidic systems were firstly used to characterize single cardiomyocytes [93,94,95], however, the concept of “heart-on-a-chip” emerged in 2011, when Grosberg et al. [96] reported a 2D anisotropic cardiac muscle tissue based on a muscular thin film (MTF) platform enabling force measurement of contractile cardiomyocytes. The device consists of arrays of thin deformable PDMS cantilevers coated with a patterned layer of fibronectin where cardiomyocytes self-organized in monolayers. The free edge of the MTF deflects vertically during systolic contraction and tissue stress is calculated based on the MTF length and its projection on the culture plane (Figure 2). In accordance to previous works [97], electrical stimulation and surface functionalization were included and improved cardiac maturation. Using optical tools along with voltage sensitive membrane dyes, Grosberg et al. also measured the action potential morphology and wavefront shape. The device was employed for pharmacological testing in a cumulative drug dose-response experiment with epinephrine. The same MTF-based heart-on-a-chip strategy was used by Agarwal et al. [98] in an aluminum-based chip. In this study, the positive inotropic effect of isoproterenol on cardiac contractility was tested, demonstrating suitability to perform rapid and accurate drug analysis. In a translational study, the MTF-based heart-on-a-chip was employed to develop a model of Barth syndrome, a mitochondrial myopathy [99]. Compared to control, Barth syndrome hiPSC-CM (human induced pluripotent stem cells-derived cardiac myocytes) cultures revealed a poor contractile function (lower twitch and peak systolic stress, reduced beating activity) and advanced pharmacological studies were carried out using bromoenol lactone, linoleic acid and arginine plus cysteine as potential therapies.

Despite the advancements in assessing cardiomyocytes contractility, MTF do not recapitulate in vivo 3D myocardial environment. In the healthy myocardium of the adult heart, cardiomyocytes are embedded in ECM and organized in a 3D brick wall-like structure, crucial to sustain a proper action potential propagation and synchronous contraction. Nunes et al. [100] made further advances on previously described in vitro anisotropic organizations of cardiomyocytes by microfabricating biowires. A PDMS culture chamber anchoring a surgical suture wire (extensively described by Sun and Nunes [101]) was used to seed hiPSC-CM and supporting cells (fibroblasts, endothelial cells and smooth muscles cells) suspended in collagen type I hydrogel. Biowires were then formed by gelation and assembled around the suture. This simple structure induced cell alignment along the suture axis and spontaneous synchronized beating was observed after 48 h of culture. Caffeine, verapamil, nifedipine and thapsigargin were used to prove that electrical stimulation promoted maturation of calcium handling mechanisms. Xiao et al. [102] used a similar biowire strategy and reported a perfusable cardiac biowire using a suspended poly(tetrafluoroethylene) tubing template, resembling native cardiac bundle. Nitric oxide (NO), which plays a critical role in myocardial function regulation, was chosen as a model drug in the biowire platform. After 24 h of NO treatment, the beating rate of cardiac myocytes slowed down, as expected, validating the platform as a biomimetic in vitro system.

Mathur et al. [103] developed a cardiac microphysiological system (MPS) constituted by a central culture chamber flanked by two adjacent medium channels. These compartments are separated by two arrays of narrow channels (2 µm). This gap distance resembles the endothelial barrier, protecting hiPSC-CM from the shear stress induced by medium perfusion while controlling nutrient diffusion. An additional weir gap at the end of the culture chamber allows cells to pack in a highly dense tissue without using exogenous cell-laden material (Figure 2). It was reported that MPS cell seeding resulted in cell alignment and spontaneous beating (50–80 beats per minute). E-4031, isoproterenol, verapamil, and metoprolol were used in pharmacological studies to validate the device as an accurate drug responsive tool. Not only hiPSC-CM beating rate response was assessed, but also half maximal inhibitory/effective concentration values (IC50/EC50) of each drug, evidencing MPS protocol as more reliable than cellular scale studies. Despite being efficaciously suitable for pharmacological studies, MPS was not equipped for applying physiologically relevant stimulations (electrical or mechanical) for training the cardiac constructs.

Marsano et al. [87] made further advances and provided a microfluidic tool that took into account broader physiological aspects in heart mimicry. The beating heart-on-a-chip was able to mechanically stimulate a cardiac construct with 10% cyclic mechanical strain, mimicking the systolic and diastolic phases that cardiomyocytes experience during the heartbeat. The microfluidic platform consisted in an upper compartment containing a central cell culture chamber and two side medium channels, and a bottom pressurized compartment. An array of hanging pillars separating both culture chamber and lateral medium channels formed a caging structure. When the pressure in the bottom chamber increases, a flexible PDMS membrane deforms, compressing the cardiac cell-laden hydrogel in the culture chamber (Figure 2). The controlled gap underneath pillars defines the stroke length for the actuation mechanism and the lateral gap between pillars confines the hydrogel solution. The distance between pillars allowed a uniaxial gel bulk elongation and spontaneous beating was observed after 2.5 days in culture. Mechanically stimulated neonatal rat cardiomyocytes, as well as hiPSC, showed higher expression of Cx43, which reflected in a synchronous contractile activity and an improved response to electrical pacing. An isoprenaline dose-response curve was constructed, validating the heart-on-a-chip for pharmacological studies. As in MPS [103], the beating heart-on-a-chip [87] used video analysis software as a non-invasive, high-throughput and cost effective approach to assess contractile performance of the cardiac tissue.

## 5. Vascular System

Due to the high control on microchannels geometrical features, the applications of microfluidics in the development of micro-vascular models has long been a subject of interest. Contrarily to in vivo models and macro-scale approaches, microfluidic channels with obstructing regions are relatively easy to produce and proved to be a high-throughput, accurate and reproducible vascular stenosis models [104,105,106]. Li et al. [104] used a branched microchannel with well-defined stenotic regions to provide insights into the effects of shear rate and antiplatelet therapy dosing on occlusion, thrombus detachment and platelet activity (Figure 2). Remarkably, the authors proved that in a high shear stress profile, eptifibatide did not reduce the occlusion time compared to control, highlighting the importance of considering hemodynamics in pharmaceutical research. Additionally, it was also shown that acetylsalicylic acid occlusion hazard was much higher compared to eptifibatide. In vitro microfluidics biomimetic vascular models also provide the understanding of how physical properties such as shear stresses can be used for the sake of local drug delivery strategies, lowering required doses, maximizing efficiency and avoiding systemic side effects [107]. The work of Korin et al. [107] led to cutting-edge advancements in organs-on-a-chip technologies for new thrombolytic drug candidates. Korin et al. used a microfluidic biomimetic vascular system able to tightly control the shear stress of stenotic regions by incorporating a narrowed channel mimicking a blood vessel with 90% lumen obstruction. Endothelial cells were cultured in the inner surface of this device to test the performance of microaggregates of poly(lactic-co-glycolic acid) nanoparticles that were engineered to breakup in regions of abnormal high shear stress (10 dyn/cm^2^). The model proved that shear-activated nanotherapeutics disruption increase in stenotic sites and, consequently, maximal endothelial cells nanoparticles uptake is achieved in the stenotic region. These findings illustrate in vitro biomimetic systems as valuable, high throughput surrogate models at the first stage of drug development.

Blood vessels barrier function is responsible for controlling the selective passage of macromolecules between blood stream and neighboring tissues in the whole body. Therefore, abnormal vascular permeability threatens homeostasis and impairs organ function and screening vasculature responses to drugs is of paramount importance. In vivo models in small animals rely on surgery-based time consuming protocols, while non-invasive techniques do not provide microscale resolution. Standard in vitro models for vascular reactivity assessment fail in mimicking anatomical structural properties and are not suitable for studies that require luminal flow. Chrobak et al. [108] provided a first in vitro model for assessing drug effects on vascular permeability. Human endothelial cell monolayers were formed on open cylindrical channels inside collagen hydrogel and showed barrier properties and immunological response. Histamine, thrombin and TNF-α successfully increased permeability [108,109] and leucocyte adhesion [108]. Likewise, Lee et al. [109] used collagen gels in engineering tubular perfusion microvessels and showed that tumor vascular hyperpermeability can be reversed in response to bevacizumab, validating this platform for potential applications in drug screening. Sato et al. [110] aspired for a more complete model and developed a highly biomimetic microcirculation-on-a-chip that, for the first time, reported the co-culture of blood vascular endothelial cells (BECs) and lymphatic endothelial cells (LECs). In this microfluidic platform, two hollow channels are partially overlapped and able to communicate through a porous polyethylene terephthalate (PET) membrane with 0.1 µm pore diameter. Histamine and habu snake venom stimulations were shown to increase permeability of BECs and LECs. These observations validate the device as an effective surrogate for testing anti-hemorrhagic potential.

Finally, Günther et al. [111] combined microfluidics with intact ex vivo artery segments to probe for artery-based pharmacological screenings. This artery-on-a-chip device is able to hold small blood vessels for long-term culture, with the advantage of fully automated acquisition of several dose-response curves. Abluminal homogeneous perfusion of acetylcholine and phenylephrine was used to assess endothelial function while different discrete regions of the artery wall were exposed to different phenylephrine concentrations proving the lack of circumferential communication. The artery-on-a-chip was also later employed as a useful tool for acquiring information on cellular organization in cerebral blood vessels [112], however, its relevance to human physiology is compromised by the usage of animal vessels.

## 6. Lung

The lungs are vital organs, responsible for gas exchanges from air to blood [113]. Since pulmonary diseases have become one of the leading causes of death, there is an urgent need to develop new strategies to quickly and consistently predict drug safety and efficacy in humans.

In the past, standard 2D static culture exploiting primary human tracheal-bronchial cells and lung primary AECs (Alveolar Epithelial Cells) seeded on multiwell or Transwell plates represented the gold standard to perform drug toxicity tests [114,115]. These monoculture systems provided basic information related to cellular responses, but lack accurate prediction of toxic drug effects due to the poor modeling of cell–cell and cell–ECM interaction [116]. Advances in microfluidic and microengineering technology provided novel approaches to generate robust lung models, mimicking the in vivo dynamical microenvironment and the essential tissue architecture [117] with the aim of performing in vitro tests that are cheaper, more accurate and unbiased by interspecies differences [118,119,120].

**Figure 3:**
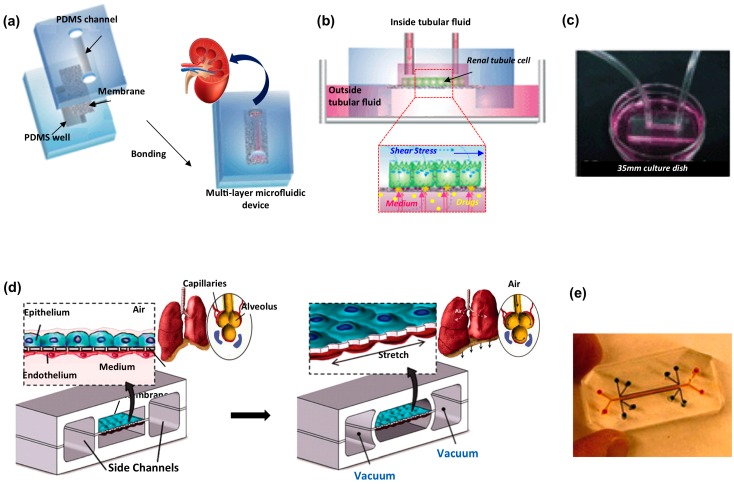
Biological barrier microfluidic models applied for kidney and lung organs. Kidney platform: (**a**) sketch of the fabrication process involving the sandwich assembly of the porous membrane between two PDMS layers; (**b**) schematic of the chip operating principle; and (**c**) picture of the device connected to a syringe pump through silicon tubing. Adapted from [137] with permission. Human lung on a chip: (**d**) schematic of the epithelial and endothelial cells co-cultured on opposite sides of the porous membrane, stretched applying vacuum in the side channels to mimic alveolar deformation during normal breathing; and (**e**) picture of a microdevice filled with color dyes. Adapted from [121] with permission.

In this perspective, great efforts have been made to reconstruct the microarchitecture of the lung alveolar-capillary interface. A pivotal example of this effort is represented by the lung-on-chip [121]. This well-known microfluidic model reproduces key functional, structural and mechanical properties of the lung functional unit: the blood-alveolar interface. It is fabricated by sandwiching two microchannels, separated by a thin, porous and flexible PDMS membrane, which allows for culturing human alveolar epithelial cells and human pulmonary microvascular endothelial cells on opposite sides (Figure 3) [121]. The model reproduces the air-liquid alveolar interface by filling the epithelial compartment with air, while keeping endothelium in contact with culture medium. Physiological breathing movements are applied to cultured cells by actuating the platform with two lateral chambers for vacuum-driven membrane stretching. The platform was exploited for analyzing the influence of physiologically relevant mechanical forces on barrier function and interactions with immune cells, pathogens, nanoparticulates, cytokines and standard or new drugs [122,123,124]. The combination of inflammatory factors (TNF-α) with physiological mechanical strain was demonstrated to activate the microvascular endothelium, promoting neutrophils adhesion and transmigration. Furthermore, it has been demonstrated that cyclic mechanical strain applied in concert with interleukin-2 (IL-2) increased the fluidic leakage through the membrane, reproducing drug toxicity–induced edema typical of cancer patients treated with this molecule [122]. Lastly, the lung-on-chip was exploited to create an airway model to analyze human lung inflammation [124]. The chip was lined with human mucociliary bronchiolar epithelium and endothelium and subjected to interleukin-13 (IL-13) to simulate hyperplasia, cytokine hypersecretion and decreased cilia functionality typical of asthmatics. In accordance with in vivo findings [125], tofacitinib suppressed cell hyperplasia, while corticosteroid resulted ineffective.

Similar asthmatic conditions were also reproduced in the previously described MTF-based device [126]. In particular, human bronchial smooth muscle (BSM) cells were organized in anisotropic laminar structures on thin films. The asthmatic condition was successfully recreated by exposing cells to interleukin-13, causing muscle hypercontractility, cellular remodeling and altering cholinergic-induced relaxation, in accordance with clinical and animal studies [127,128]. Notably, the authors employed this model to assess the efficacy of a newly proposed drug (HA1077) in treating acute bronchoconstriction.

Aside from platforms reproducing alveolar-capillary interfaces [122,123,124,129] or musculature [126], another family of devices specifically designed for high-throughput screening of new anti-lung cancer candidates is here described. Wang et al. developed an integrated microfluidic device composed by an upstream concentration gradient generator (CGG) and downstream cell culture chambers to induce different level of glucose regulated proteins (GRP78) expression in human lung squamous carcinoma cell line cultured as monolayers [130]. Findings include that cellular apoptosis increased in a dose dependent manner, while a subsequent treatment with anti-cancer drug (etoposide VP-16) decreased cell death, demonstrating GRP78 plays a key role in anti-cancer drugs resistance. A similar approach was exploited by Ying et al. [131], who developed an integrated microfluidic platform with a linear CGG network to demonstrated that hepatocyte growth factor from cancer-associated fibroblasts up-regulates GRP expression in human non-small cell lung cancer A549, inhibiting paclitaxel-induced cell apoptosis. In both studies a CGG was successfully exploited to test different molecule concentrations in parallel, however, in the latter study cells were cultured in a 3D environment, thus better mimicking in vivo cellular pharmacological resistance [132,133]. Lastly, it is worth noting other microfluidics approaches that, in addition to the dimensionality of cultured cells, took into account environmental stimuli such as hypoxia [134], constant perfusion [135] or co-cultures [136] to better study drug resistance of lung-associated tumors.

## 7. Kidney

The kidney is a highly organized organ containing more than ten types of renal cells structured in a 3D network around a complex vasculature system [138]. The renal basic structural and functional unit is the nephron, composed by the glomerulus and the tubule that are responsible for filtration of solutes and for secretion/reabsorption of active molecules, respectively [139].

Nephrotoxicity and drug-induced kidney injury (DIKI) represent important dose-limiting factor in pharmacotherapy often detected only during later phases of drug development (clinical trials and post approval) [140,141,142]. Relevant in vitro kidney models are needed to better predict molecular mechanism of human drug toxicity and to be successfully exploited in early drug screening processes. However, it is known that currently available models (tissue slices or kidney cell lines cultured in Petri dishes) fail to fully recapitulate the biological functions of the kidney cells resulting in poor prediction of DIKI [143,144]. On the other hand, studies performed under environmental cues such as physiological fluid shear stress (FSS) demonstrated that renal cells better develop and maintain in vivo-like phenotypic characteristics, organization and performances [145,146,147].

Microfluidic technologies are candidate for precisely tailoring the in vivo-like microenvironmental cues including FSS and to reconstitute the original renal cell phenotype, leading to increased sensitivity to toxic agents and more reliable screenings [148]. We report a significant number of studies that still involves the use of animal-derived cells. In this context, Baudoin and colleagues successfully demonstrated the potential of using a constant perfusion microfluidic platform [149] for drug toxicity studies on Madin Darby Canine Kidney (MDCK) cells [150]. The application of FSS enhanced cellular proliferation and re-organization in a 3D tissue, while the administration of ammonium chloride decreased cell proliferation rate and induced morphological changes, as previously reported in literature [151]. In the same platform, the dynamic condition was also found to enhance MDCK cell functions, in terms of typical kidney transporters up-regulation (e.g., calcium, phosphate, sodium, and H+ protons homeostasis) [144]. In another study, Chouca Snouber et al. exploited this platform and demonstrated that cells treated with the anti-neoplastic ifosfamide compound showed a reduction in inflammatory responses, that was not evidenced on cells cultured in standard cell culture systems and subjected to the same treatment [152]. These results were of particular interest, demonstrating the potential of microfluidic platform in determining the bridge connecting inflammation and tumors [153].

In addition to perfusion-based systems, pertinent blood-kidney barrier models are fundamental to better mimic cellular filtering capacity and to better investigate molecular transport phenomena. Traditional Transwell systems have thus been fully exploited to study cross-epithelial transport [154,155], but for the static nature of the model cells lack functional differentiation. Microfluidics offers the possibility to combine the barrier model with specific pattern of FSS stimulation, improving cellular phenotype and functionality and leading to a better barrier model [137,156,157]. In this context, Jang et al. demonstrated that the fluidic shear of 1 dyn/cm^2^ applied for 5 h on primary rat inner medullary collecting duct (IMCD) cells cultured on a porous membrane within a double-layer microfluidic device enhanced cellular conformation rearrangement (highly polarized columnar morphology vs. flattened form), cytoskeletal reorganization and cell junctions [137]. With this platform, the authors reproduced the tubule function, demonstrating that cells successfully regulate water and ion balance under hormonal stimulation (Figure 3). With the same approach, Ramello et al. developed a microfluidic platform to study the filtration capability of the glomerulus, demonstrating that acrolein increased the solutes mass transport in a dose-dependent manner [157]. The platform consisted of a filtration unit composed by two PDMS compartments reproducing the glomerular apical and basolateral sides, separated by a porous membrane seeded with MDCK cells, across which the transport phenomena occurred. Kim and colleagues [158] exploited a similar platform to analyze not only the influence of a drug on the barrier integrity, but also its administration regimen. The authors demonstrated that gentamicin increases cell injury and barrier impairment more consistently when MDCK cells are continuously exposed to the antibiotic agent in a dynamic culture within the microfluidic device. On the contrary, in Transwell systems, short-term high concentration regimen resulted more cytotoxic than long-term low concentration exposure. This finding is coherent with other publication results [159] and is mainly related to the fact that renal tubule cells exposed to physiological shear stress retain native cell polarization and enhance tight junctional protein expression improving epithelial barrier stability [145,156,160]. This model holds promise for finding more efficient drug administration protocols in clinical trials, that today are based on animal tests [161] poorly predicting human drug toxic effect because of higher animal renal clearance [162] and inter-species differences in drug transporters and metabolism.

Although these microfluidic devices recapitulated key features of the kidneys, they are limited by the use of animal-derived cells. Significant differences indeed exist between animals and humans in terms of substrate specificity, expression, relative abundance and tissue distribution of clinically relevant cellular drug transporters in the kidney [163,164]. This observation pushed Jang et al. to seed a porous membrane-based microfluidic device [137] with human proximal tubule epithelial cells (HPTEC) to study nephrotoxic effect of cisplatin in the so-called proximal-tubule-on-a-chip [165]. Confirming the results previously reported in animal cells, HPTEC exposed to human physiological shear stress (0.2 dyn/cm^2^) restored normal columnar form, increased expression of Na/K-ATPase, aquaporin 1 (AQP1) water channels and cross epithelial transporters fundamental for the proximal tubule functionality. In addition, FSS enhanced the number of cells expressing primary cilia, key component in mechanosensing and tubular morphology regulation. The authors exploited this model to study nephrotoxic effects of the chemotherapeutic cisplatin, demonstrating lower cellular injury and more incisive recovery effect of cimetidine, compared to Transwell culture systems.

Human in vitro models therefore represent potent tools for nephrotoxicity screening, reproducing relevant kidney functions thanks to the combination of suitable cell systems and microfluidic culturing technologies. Furthermore, kidney-on-chip devices can also be exploited to establish new quantitative, sensitive and clinically relevant DIKI biomarkers to predict cellular toxicity after drug exposure. As demonstrated by Adler et al. [166], HPTEC cells cultured within a 3D microfluidic chip [167] and exposed to cadmium chloride overexpressed a previously defined sensitive biomarker [166], highlighting the possibility to perform high-throughput quantitative screening of nephrotoxic compounds in microfluidic chips. Finally, we highlight a recent human kidney proximal tubule microphysiological system where human renal cells were arranged in a structural organization remarkably similar to in vivo structures and exhibiting physiological renal functions [168].

## 8. Perspective Outlook: Body-on-Chips

In this review, we highlighted microfabricated culture systems employed for drug-screening applications. We focused on studies that, by applying a variety of microfabrication strategies for improving the mimicry of organ-level or tissue-level environments in vitro, envision a more rational and physiologically relevant pre-clinical drug screening in vitro testing. Current literature is not limited to the biological structures covered in the previous paragraph, and it is worth mentioning that studies also report microfluidics-based drug screening for skeletal muscle tissue [169], cartilage [170], retina [171], and spleen [172]. We highlighted how part of the described studies employed human-derived cells, thanks to the low volumes required for seeding and culturing microdevices. In this respect, hiPSC cells are expected to represent better human models in the future but still require optimization of protocols for fully exploiting their differentiation potential. Limitations and drawbacks affect this class of microdevices and need to be addressed. The most popular polymer for microfabrication (PDMS) faces general challenges in terms of scale-up production for commercialization especially when the device complexity increases (e.g., insertion of electrodes in heart- or CNS-related devices; insertion of porous membranes in kidney- or CNS-related devices). Geometrical limitations may also affect, for instance, finely designed vascular-mimicking devices (circular sections below 25 μm cannot be achieved [173,174]). In addition, PDMS is known to absorb hydrophobic compounds therefore limiting and affecting the use of drug candidates.

In this scenario, it is also tempting to devise modular approaches where multiple biological structures are cultured on a single device and challenged with a candidate drug compound in a single experiment. Body-on-chip devices would represent unique tools, created by incrementally include interactions between multiple organs or tissue units in one device, to predict organism-level pharmacokinetics and pharmacodynamics of candidate drug compounds before human clinical trials. Indeed, pioneering works and perspective papers are present in current literature discussing and implementing modular solutions [175,176,177,178,179,180,181]. Maschmeyer et al., report a four-organ microdevice where an on-chip pumping system interconnects intestine, skin, liver and kidney cell culture modules. In addition, a second microfluidic circuit is deputed to kidney module fluid drainage and collection [175]. Cultures were viable for as long as 28 days and specific gene expression analyses confirmed the functionality of single compartments. However, no drug stimulation experiment was performed and the design lacks clear scaling criteria.

This perspective class of microdevices faces many challenges, of which the definition of accurate design criteria is the initial and paramount task. In order for body-on-a-chip devices to accurately predict organism-level drug response, pharmacokinetics and/or pharmacodynamics models need to be defined and adapted to candidate chip designs [182]. In this respect, size scaling of the biological units, residence time, flow rates of the drug compounds within a specific biological unit and interactions between functional units in multi-compartment devices are key parameters to be taken into account for rational coupling of device design and pharmacokinetics/pharmacodynamics modeling. Wikswo et al. discussed scaling of biological units within multi-organ microfluidic devices and proposed functional organ parameters for basing scaling factors [183]. Moreas et al. reviewed scaling strategies proposing metabolic rate as an ideal parameter for scaling purposes [184]. Shuler et al. also reviewed and discussed rational body-on-chip device design and firstly report a set of design criteria that parametrically define relevant microfluidic device variables such as concentrations and flow rates per each compartment. In this last example, mean drug residence time within specific organs is used to scale the compartments [185,186]. In addition to scaling criteria, the definition of a universal cell culture medium is also a fundamental open task.

In summary, with more than a hundred reported drug/chemical screening applications, microfabricated strategies represent a potentially relevant approach for directing in vitro pre-clinical studies. Nevertheless, most of the papers here reviewed employ drug screening as a validation step for newly developed microplatforms. There is a need for in-depth studies performed with standard procedures and providing novel insights into in vitro drug safety, efficacy and mechanisms of action to further prove capability of microfluidic-based biological models and to effectively give rise to a new generation of drug screening microdevices.
